# Advancements in understanding cardiotoxicity of EGFR- TKIs in non-small cell lung cancer treatment and beyond

**DOI:** 10.3389/fphar.2024.1404692

**Published:** 2024-08-15

**Authors:** Xin Li, Yongping Lin, Song Lin, Jiayi Huang, Zhongbao Ruan

**Affiliations:** Department of Cardiology, The Affiliated Taizhou People’s Hospital of Nanjing Medical University, Taizhou School of Clinical Medicine, Nanjing Medical University, Taizhou, China

**Keywords:** cardiotoxicity, epidermal growth factor receptor-tyrosine kinase inhibitors, cardio-oncology, non-small cell lung cancer, cardiovascular toxicity

## Abstract

Epidermal Growth Factor Receptor-Tyrosine Kinase Inhibitors (EGFR-TKIs) are a class of oral targeted anticancer drugs that have been demonstrated to significantly inhibit tumor progression and improve clinical prognosis in patients diagnosed with EGFR-mutated tumors, particularly in those with non-small cell lung cancer. However, the sustained usage of EGFR-TKIs may cause potential cardiotoxicity, thus limiting their applicability. The primary objective of this review is to systematically analyze the evolving landscape of research pertaining to EGFR-TKI-induced cardiotoxicity and elucidate its underlying mechanisms, such as PI3K signaling pathway inhibition, ion channel blockade, oxidative stress, inflammatory responses, and apoptosis. Additionally, the review includes an exploration of risk assessment for cardiotoxicity induced by EGFR-TKIs, along with management and response strategies. Prospective research directions are outlined, emphasizing the need for more accurate predictors of cardiotoxicity and the development of innovative intervention strategies. In summation, this review consolidates recent research advances, illuminates the risks associated with EGFR-TKI-induced cardiac toxicity and presents crucial insights for refining clinical dosage protocols, optimizing patient management strategies, and unraveling the intricate mechanisms governing EGFR-TKI-induced cardiotoxicity.

## 1 Introduction

Tyrosine kinases constitute a family of proteins that, upon activation, catalyze the phosphorylation of tyrosine residues on other intracellular proteins (Gocek et al., 2014). Epidermal Growth Factor Receptor-Tyrosine Kinase Inhibitors (EGFR-TKIs) function as competitive inhibitors of EGFR-TK activation, displaying high affinity for the adenosine triphosphate (ATP) binding site of EGFR. Consequently, they competitively bind to the intracellular tyrosine kinase domain of EGFR, inhibiting ATP from binding to its site. This disruption of ATP binding leads to the inhibition of receptor autophosphorylation, thereby obstructing downstream signaling. Ultimately, EGFR-TKIs impede tumor growth, proliferation, and differentiation, inducing apoptosis in tumor cells (Chaar et al., 2018; Harrison et al., 2020).

EGFR-TKIs is indicated for patients with a diverse array of tumors carrying EGFR mutations, encompassing non-small cell lung cancer (NSCLC), pancreatic cancer, glioblastoma, and head and neck squamous cell carcinoma (Mukasa et al., 2010; Wang et al., 2024; Zhang et al., 2021). Its most prevalent application is in the management of NSCLC which accounts for approximately 85% of all lung cancer cases (Alanazi et al., 2021). The survival rate of NSCLC varies depending on the stage of the disease at diagnosis (Siegel et al., 2012). If detected early, when the tumor is still confined to the lungs, the 5-year survival rate can be as high as 56%. However, most patients are diagnosed at later stages (Majeed et al., 2021), such as when the tumor has spread to other organs, at which point the 5-year survival rate may drop to as low as 5% (Gettinger and Lynch, 2011). Additionally, the progression rate of NSCLC can range from relatively slow to extremely rapid, depending on the biological characteristics of the tumor and its molecular markers (Yuan et al., 2019). In the Chinese NSCLC population, approximately 30% of patients exhibit EGFR mutations, showcasing a significant impact of EGFR-TKIs on this subgroup (Shi et al., 2014). Currently, three generations of EGFR-TKIs are in clinical use: the first generation includes Gefitinib, Erlotinib, and Icotinib; the second generation comprises Afatinib, Dacomitinib, and Lapatinib; the third generation encompasses Osimertinib, Almonertinib (Aumolertinib), Alflutinib (Furmonertinib), and Lazertinib (Table 1). The fourth-generation EGFR-TKIs such as EAI045, TQB3804, and JNJ-61186372 (JNJ-372) are either in development or undergoing clinical trials. Notably, the third-generation EGFR-TKIs have successfully overcome resistance observed with first- and second-generation counterparts. They act as irreversible EGFR inhibitors, effectively managing cancer progression induced by EGFR-TKI-sensitive and T790M-resistant mutations, with an added advantage of controlling brain metastases (Cross et al., 2014; Remon and Planchard, 2015; Anand et al., 2019). While monotherapy with EGFR-TKIs has demonstrated superior efficacy and tolerability compared to standard chemotherapy, a notable drawback is their association with cardiac toxicity (Chang et al., 2023). The concurrent manifestation of cardiotoxicity frequently necessitates the temporary or premature discontinuation of anticancer therapy in patients undergoing targeted treatment (Ikebe et al., 2020). Striking a balance between the benefits of targeted cancer therapy and the cardiovascular risks has becoming a pressing and intricate concern for clinicians (Schiefer et al., 2018).

**TABLE 1:** Cardiotoxicity description of EGFR-TKIs.

Generation	Name	Targets	Cardiotoxicity
First Generation	Gefitinib	EGFR, EGFR21 exon L858, EGFR-Ex19del	MI (Yamaguchi et al., 2005), cardiomyopathy (Korashy et al., 2016; AlAsmari et al., 2020), prolonged QT interval (Waliany et al., 2021), thromboembolism (Lynch et al., 2011)
	Erlotinib	EGFR, EGFR21 exon L858R, EGFR-Ex19del	MI (Kus et al., 2015), ischemia, cardiomyopathy (Pinquié et al., 2016), prolonged QT interval (Waliany et al., 2021), decreased LVEF (Noma et al., 2007; Nagashio et al., 2021)
	Icotinib	EGFR, EGFR21 exon L858R, EGFR T790M, EGFR L861Q	Palpitation, hypotension
Second Generation	Afatinib	EGFR, EGFR21 exon L858, EGFR-Ex19del, HER2, HER4	Pericardial effusion (Nuvola et al., 2019), cardiomyopathy (Ramos et al., 2022)
	Dacomitinib	EGFR, EGFR2, EGFR4	——
	Lapatinib	EGFR HER2	HF (Huijberts et al., 2020), asymptomatic decreased LVEF, prolonged QT interval (Ando et al., 2020)
Third Generation	Osimertinib	EGFR21 exon L858, EGFR-Ex19del, EGFR T790M, HER2	Prolonged QT interval (Schiefer et al., 2018; Waliany et al., 2021; Li et al., 2023), HF (Ikebe et al., 2020), pericardial effusion, AF (Anand et al., 2019), MI (Kunimasa et al., 2020), cardiomyopathy (Patel et al., 2023), cardiac valvular lesions (Kunimasa et al., 2020)
	Almonertinib	EGFR21 exon L858, EGFR-Ex19del, EGFR T790M	Prolonged QT interval, HF, asymptomatic decreased LVEF, arrhythmia
	Lazertinib	EGFR21 exon L858, EGFR-Ex19del, EGFR T790M	Prolonged QT interval, decreased LVEF

EGFR, epidermal growth factor receptor; MI, myocardial infarction; LVEF, left ventricular ejection fraction; HF, heart failure; AF, atrial fibrillation.

## 2 Mechanisms of cardiotoxicity caused by EGFR-TKIs

While the precise mechanism underlying cardiotoxicity induced by EGFR-TKIs remains incompletely understood, existing studies offer a basis for exploring potential mechanisms from various perspectives.

### 2.1 Inhibition of the PI3K signaling pathway and effects on ion channels

The fast-activating delayed rectifier K^+^ current (IKr), mediated by the K^+^ channel encoded by the human ether-a-go-go-related gene (hERG), constitutes the principal component of cardiac repolarization during the third phase of the cardiac action potential, particularly in large animals, including humans. Pharmacological blockade of the cardiac potassium channel of hERG leads to a decrease in IKr, which is a crucial molecular mechanism contributing to the prolongation of QT intervals and, consequently, the initiation of cardiac arrhythmias (Yang and Nerbonne, 2016; Chiamvimonvat et al., 2017; Helliwell et al., 2023). Intriguingly, some drugs that induced long QT syndrome by directly blocking IKr also exhibit inhibitory effects on the PI3K signaling pathway, a phenomenon that may contribute to their arrhythmogenic potential (Yang et al., 2014; Ballou et al., 2015).

Many TKIs, such as nilotinib, vandetanib, erlotinib, gefitinib, and imatinib, have been demonstrated to inhibit hERG currents by directly blocking hERG channels and/or modulating acute effects on channel gating (Kopljar et al., 2018; Jie et al., 2021; Cui et al., 2022; Li et al., 2023). Additionally, certain TKIs, including erlotinib, can hinder the phosphatidylinositide 3-kinases-Serum and glucocorticoid-regulated kinase 1 (PI3K-SGK1) signaling pathway, disrupting the post-transcriptional process of hERG potassium channel functional protein. This interference promotes the internalization of mature protein on the membrane, leading to reduced membrane hERG channel protein expression, further inhibiting hERG current, and resulting in QT interval prolongation (Cui et al., 2022).

Experimental studies involving canine cardiomyocytes treated with either a TKI or a PI3K inhibitor demonstrated an increase in action potential duration, while the intracellular infusion of phosphatidylinositol 3,4,5-triphosphate, a downstream effector of the PI3K pathway, reversed this effect. This suggests that the inhibition of PI3K-SGK1 signaling contributes to the prolongation of action potential duration and QT interval (Lu et al., 2012).

Insulin receptor tyrosine kinase activates PI3Kα to produce phosphatidylinositol 3,4,5-trisphosphate, recruiting protein kinase B (Akt) and phosphatidylinositol 3-phosphate-dependent protein kinase 1 (PDK1) to the plasma membrane, leading to Akt activation. Receptor tyrosine kinase also activates atypical protein kinase C and SGK *via* PDK1. Akt, PDK1, atypical protein kinase C, SGK, and other downstream effectors of PI3K may regulate ion channels conducting potassium, sodium, and calcium current (Ballou et al., 2015). Zhang et al. proposed that the regulation of PI3K/Akt pathway inhibition and its downstream molecules, resulted in an increase in persistent sodium current and a decrease in L-type calcium current, potassium current (IKr and IKs), ultimately leading to QT interval prolongation (Zhang et al., 2023).

**FIGURE 1:**
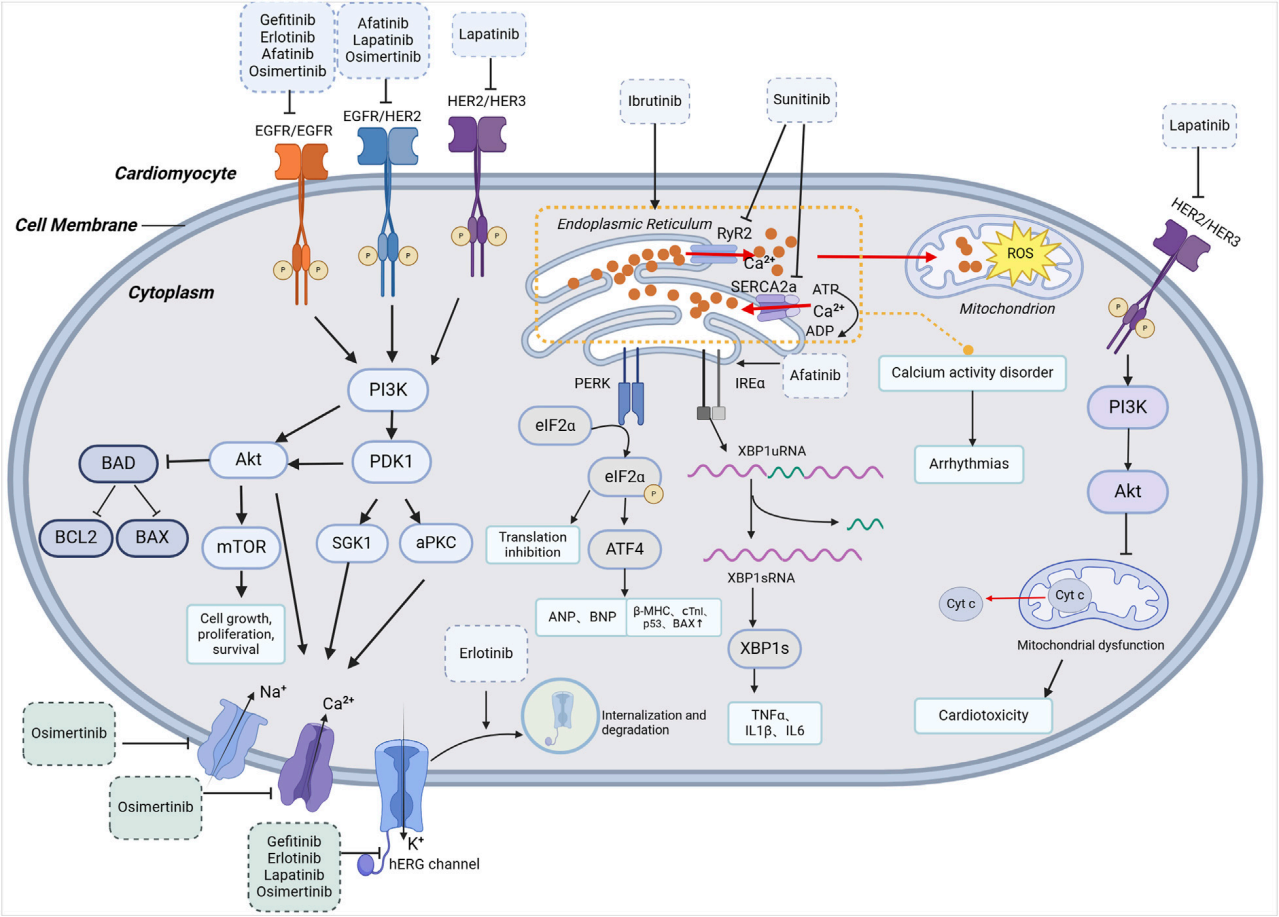
Mechanisms related to cardiotoxicity of EGFR-TKIs. PI3K, phosphatidylinositide 3-kinases; Akt, recruiting protein kinase B; PDK1, phosphatidylinositol 3-phosphate-dependent protein kinase 1; mTOR, mammalian target of rapamycin; BAD, Bcl-xL/Bcl-2asociated death promoter; BCL2, B-cell lymphoma-2; BAX, Bcl-2-associated X protein; PERK, Protein kinase R (PKR)-like endoplasmic reticulum kinase; IREα, Inositol requiring enzyme α; eIFα, eukaryotic initiation factor α; ATF4, activating transcription factor 4; XBP1, X-Box Binding Protein 1.

In a 2023 study, Peiwen Li’s team found that osimertinib prolonged QT interval, PR interval, QRS complex, and left atrial, left ventricular, and atrioventricular conduction time in isolated guinea pig hearts. Osimertinib also concentration-dependently blocked hERG, Nav1.5, and L-type Ca^2+^ channels. Therefore, they suggested that the cardiotoxic effects of osimertinib, such as QT interval prolongation and the reduction of Left Ventricular Ejection Fraction (LVEF), were attributed to its effects on hERG, Nav1.5, and L-type Ca^2+^ channel (Li et al., 2023). Different EGFR-TKIs may block various ion channels, but the primary ion channels they affect seem to be hERG potassium ion channels. For example, lapatinib predominantly blocks hERG channels, mildly affecting IKs current, while exhibiting no blocking effect on sodium current, inward rectifier potassium current, and calcium current (Zhang et al., 2023). In conclusion, compared to direct channel blocking, the final QT prolongation may be caused by inhibition of the PI3K/Akt signaling pathway and consequent changes in ion current (Figure 1).

### 2.2 Inhibition of HER2

The well-established EGF family comprises four members, discovered in sequential order: EGFR, also known as human epidermal growth factor receptor 1 (HER1, ERBB1), human epidermal growth factor receptor 2 (HER2, ERBB2), human epidermal growth factor receptor 3 (HER3, ERBB3), and human epidermal growth factor receptor 4 (HER4, ERBB4) (Kwapiszewski et al., 2016). HER2 is expressed across multiple organ systems, including the cell membrane of cardiomyocytes, playing a crucial role in cardiomyocyte survival, growth, and stress response (Lenneman and Sawyer, 2016). Its related pathway, the ErbB/neuregulin 1 (NRG1) pathway, stands as a significant mechanism facilitating cardiac adaptation to stress (Odiete et al., 2012; Belmonte et al., 2015; Grego-Bessa et al., 2023). NRG1 activates ErbB2 phosphorylation and intracellular signaling through PI3K/Akt and ERK1/2 pathways. Its downstream signaling involves the upregulation of glutathione reductase and endothelial nitric oxide synthase mRNA, leading to decreased production of reactive oxygen species (ROS). This suggests that ErbB2/NGR1 signaling pathway plays a certain role in the antioxidant defense of cardiomyocytes (Timolati et al., 2006; Eldridge et al., 2014; Leemasawat et al., 2020). The existing research indicates that TKIs are involved in this pathological pathway. Lapatinib exhibits cardiotoxicity by diminishing ErbB2 phosphorylation. Furthermore, research has identified that osimertinib and afatinib can impair HER2 function while inhibiting EGFR, thereby inducing cardiac dysfunction (Figure 1) (Li et al., 2008; Cross et al., 2014; Herrmann, 2020).

### 2.3 Oxidative stress, ER stress, and inflammatory responses

Huan Wang et al. performed a comprehensive analysis of eight TKIs exhibiting varying degrees of cardiotoxicity, assessing the phenotypic and transcriptomic responses in human cardiomyocytes subjected to different doses and durations of TKIs. Among them, afatinib, sorafenib, and ponatinib showed the ability to activate the protein kinase R-like sarcoplasmic reticulum kinase or inositol requiring enzyme 1α axis of the endoplasmic reticulum stress (ERS) pathway, leading to ERS induction and cardiac fetal gene expression, ultimately causing cardiotoxicity (Wang et al., 2023). TKI-induced ERS upregulated inflammation in cardiomyocytes, involving the inflammasome-IL1β pathway and the nuclear factor kappa-B pathway. This presents a potential therapeutic target for attenuating sorafenib- and ponatinib-induced cardiotoxicity (Figure 1). However, in the case of afatinib, ERS activation is essential for drug-induced apoptosis in tumor cells. Inhibiting ERS might adversely affect afatinib’s therapeutic efficacy, making it an unsuitable candidate for treating TKI-related cardiotoxicity. The study also revealed that afatinib induced varying degrees of lipid peroxidation and an increase in ROS in rat cardiomyocytes (Wang et al., 2023). Schreier et al. discovered that targeted deletion of EGFR in vascular smooth muscle isolated from a mouse aorta led to spontaneous cell death, implicating oxidative stress in this process (Schreier et al., 2011). Furthermore, EGFR-TKIs with concurrent HER2 inhibition were associated with a concentration-dependent increase in ROS production (Fox et al., 2020).

### 2.4 Autophagy and apoptosis in cardiomyocytes

Gefitinib treatment of rat cardiomyocytes demonstrated a time- and concentration-dependent upregulation of hypertrophic gene markers at both mRNA and protein levels, including BNP and β-myostroponinn heavy chain. Concurrently, anti-hypertrophic gene markers were downregulated, resulting in an increased proportion of hypertrophic cardiomyocytes. Additionally, there was a proportional and concentration-dependent elevation in the mRNA and protein levels of apoptotic markers, such as troponins I, p53, and Bcl-2-associated X protein, strongly indicating that gefitinib initiates events leading to apoptotic death in cardiomyocytes (Figure 1) (Korashy et al., 2016; AlAsmari et al., 2020). Lapatinib treatment increased the ratio of pro-apoptotic BCL-Xs to anti-apoptotic BCL-Xl protein, potentially causing ATP depletion and inducing cardiomyocyte apoptosis (Hervent and De Keulenaer, 2012). In 2020, Ali Alhoshani et al. observed an increase in the level of LC3II protein, a marker of autophagy, in mouse cardiomyocytes following gefitinib treatment (Alhoshani et al., 2020). Monika E. Grabowska’s team, in 2021, found that sorafenib, sunitinib, ponatinib, trastuzumab, and gefitinib induced high levels of apoptosis, while nilotinib and erlotinib induced lower levels of apoptosis based on a computational model of cardiac apoptosis that they constructed and validated. The reduced contractility of neonatal rat 3D engineered heart tissue after the administration of sunitinib, imatinib, sorafenib, and lapatinib was associated with impaired autophagy function and the presence of autophagic lysosomes in ultrastructural evaluation (Grabowska et al., 2021). Zhang Z et al. discovered that osimertinib could induce autophagy by inhibiting the PI3K/Akt signaling pathway (Zhang et al., 2019). It has also been suggested that the inhibition of targeted ErbB2 can alter Bcl-x splicing and induce endogenous apoptotic signals, leading to apoptosis and triggering cardiotoxicity (Rohrbach et al., 2005; Yeh and Bickford, 2009).

### 2.5 Calcium activity disorder

In cardiomyocytes, intracellular calcium transients integrate electrochemical signals from the action potential with molecular signaling pathways that regulate contraction, and disruptions in intracellular Ca^2+^ transients are associated with many arrhythmias (Bers, 2002; Altamirano and Bers, 2007; Lu et al., 2015; Kopljar et al., 2018; Du et al., 2020; Zhu et al., 2022). Ca^2+^ also plays a crucial role in maintaining sarcoplasmic reticulum homeostasis and facilitating cardiomyocyte contraction, while Ca^2+^ overload is associated with ERS and systolic/diastolic defects (Frisk et al., 2021; Wang et al., 2023). According to Jong J. Kim, drug-induced ventricular arrhythmia in patients with type 2 long QT are underlain by synchronized systolic subcellular Ca^2+^ elevation (Kim et al., 2015). Beyond long QT syndrome, abnormalities in intracellular free calcium have been implicated in various pathologies (Bers, 2014; Dridi et al., 2020; Hutchings et al., 2022). In human and animal models of heart failure (HF), the reduced amplitude and slower attenuation of intracellular free Ca^2+^ transients, stemming from diminished expression/function of sarcoplasmic reticulum Ca^2+^-ATPase, are often accompanied by increased Na^+^/Ca^2+^ exchanger activity and decreased inward rectifier potassium current amplitude. In these scenarios, residual adrenergic activity might induce sarcoplasmic reticulum Ca^2+^ overload, triggering spontaneous Ca^2+^ release events and initiating arrhythmias (Pogwizd et al., 2001; London et al., 2003). Spontaneous Ca^2+^ release through the cardiac ryanodine receptor (RyR2), termed “storage overload-induced Ca^2+^ release”, is a common mechanism of arrhythmia. Arrhythmias induced by silmitasertib (a casein kinase 2 inhibitor) and sunitinib have been reported to result from their effects on RyR2 and increased storage overload-induced Ca^2+^ release (Figure 1) (Chakraborty et al., 2019). Although EGFR-TKIs such as osimertinib may have a similar effect, further studies are necessary to confirm this hypothesis.

### 2.6 Mitochondrial dysfunction

Lei Sun et al. conducted experiments using a combination of lapatinib and doxorubicin to treat cardiomyocytes. The results showed that lapatinib enhanced oxidative stress and ferroptosis in cardiomyocytes by inhibiting the PI3K/Akt pathway, which ultimately resulted in mitochondrial dysfunction. This exacerbation of doxorubicin-induced cardiotoxicity was linked to lapatinib’s impact on cellular processes (Sun et al., 2022). Numerous studies have demonstrated that HER2 inhibition can induce mitochondrial dysfunction and disrupt adequate energy supply (Grazette et al., 2004; Gordon et al., 2009). While lapatinib did not exhibit a significant effect on nitric oxide synthase expression and basal mitochondrial respiration, it did impair the reserve oxygen consumption rate (Figure 1) (Rana et al., 2012; Hsu et al., 2018). These findings suggested that lapatinib’s interference with mitochondrial function might contribute to the exacerbation of doxorubicin-induced cardiotoxicity, potentially through the disruption of cellular energy homeostasis.

### 2.7 Other possible mechanisms of cardiotoxicity

The study conducted by Huan Wang et al. also revealed that afatinib induced the deletion of TNNT2 protein, known as cardiac troponin T2, a vital component of the myocardial sarcomere and a marker of myocardial injury. The diminished expression of this protein may have adverse effects on cardiomyocyte contraction, suggesting a potential mechanism for afatinib-induced cardiotoxicity (Wang et al., 2023). Gefitinib was found to significantly enhance platelets’ ability to produce thromboxane A2, thereby elevating their prothrombotic capacity (Kanazawa et al., 2006; Lynch et al., 2011; Zaborowska-Szmit et al., 2016). Additionally, it has been proposed that gefitinib, through its inhibition of epidermal growth factor, might render the surface of atherosclerotic plaques more susceptible to damage, potentially contributing to cardiotoxicity (Miyagawa et al., 1995; Kawakami et al., 2003; Zaborowska-Szmit et al., 2020). Chronic erlotinib treatment, as reported by Mak et al., led to hypomagnesemia, triggering substance P receptor-mediated oxidative stress and resulting in cardiac dysfunction (Mak et al., 2015). Takahisa Noma’s findings suggested that β-inhibitor-mediated trans-activation of the β1-adrenergic receptor EGFR confers cardioprotection. In a mouse model of catecholamine overdose, EGFR signaling itself exhibited a protective effect (Noma et al., 2007). Pharmacologic inhibition of EGFR by erlotinib resulted in dilated cardiomyopathy after chronic catecholamine infusion in mice. Consequently, in the presence of catecholamine overproduction, erlotinib may impede myocardial protective signaling by inhibiting EGFR, ultimately leading to HF (Noma et al., 2007; Nagashio et al., 2021).

## 3 Clinical manifestations of EGFR-TKIs-related cardiotoxicity

**FIGURE 2:**
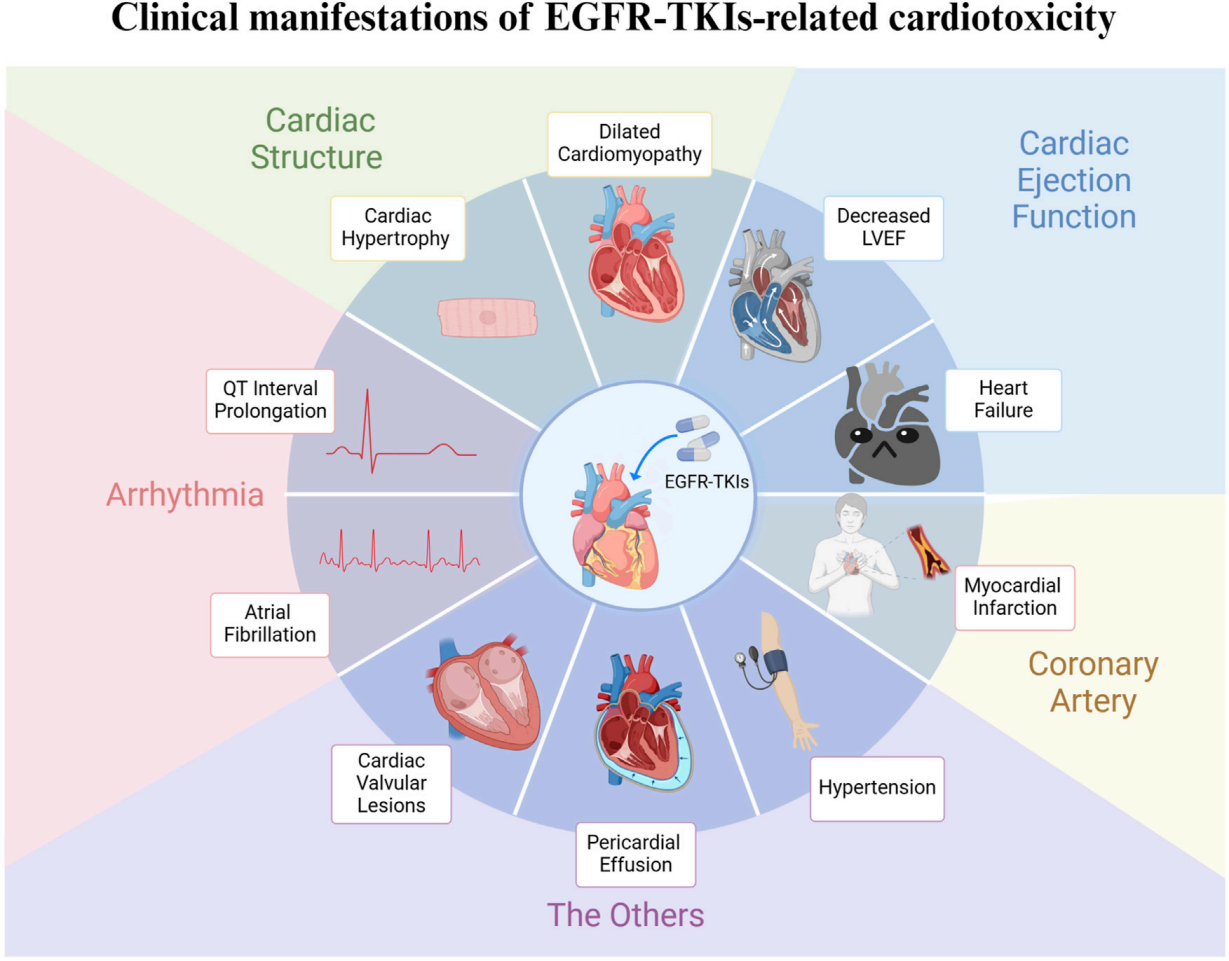
Clinical manifestations of EGFR-TKIs-related cardiotoxicity. The toxic effects of EGFR-TKIs on the heart have different clinical manifestations, which can be roughly divided into the following aspects. Effects on cardiac structure: myocardial hypertrophy, dilated cardiomyopathy; The effects on cardiac ejection function: decreased left ventricular ejection fraction (LVEF), heart failure; The effects on cardiac rhythm: QT interval prolongation, atrial fibrillation, etc.; The effects on coronary artery: myocardial infarction; Others: pericardial effusion, cardiac valvular lesions, hypertension, etc.

Since the advent of EGFR-TKI drugs, their associated cardiovascular adverse events have been extensively documented, including prolonged QT intervals, HF, atrial fibrillation (AF), myocardial infarction (MI), pericardial effusion, hypertension, and cardiac valvular lesions (Figure 2) (Lenneman and Sawyer, 2016; Kenigsberg et al., 2017; Chitturi et al., 2022).

### 3.1 QT interval prolongation

Extended QT intervals pose an elevated risk for severe arrhythmias, including torsade de pointes (TdP), a potentially fatal condition precipitating sudden cardiac death (Davies et al., 2023). Sarah Waliany assessed the cardiovascular adverse event risk associated with EGFR-TKIs utilizing the WHO pharmacovigilance database VigiBase. The investigation, spanning different categories of TKIs deployed in NSCLC-targeted therapies, revealed that EGFR-TKIs escalated the odds of QT interval prolongation relative to the complete database (ROR = 1.39, 99% CI: 1.08–1.80, IC025 = 0.18). Intriguingly, when contrasted with other targeted therapies, EGFR-TKIs demonstrated a decreased likelihood of QT interval prolongation (ROR = 0.26, 99% CI: 0.19–0.35).

Within the realm of EGFR-TKIs, the third-generation variant, osimertinib, exhibited substantially higher rates of QT interval prolongation compared to other EGFR-TKIs (ROR = 49.19, 99% CI: 25.88–93.47), other targeted NSCLC therapies (ROR = 6.13, 99% CI: 4.43–8.48), and the overall VigiBase database (ROR = 14.34, 99% CI: 10.79–19.04, IC025 = 3.38). Importantly, osimertinib manifested significantly increased odds of QT interval prolongation, with a median onset of 29.3 days, suggesting a robust correlation between osimertinib use and QT interval prolongation, with a 49-fold higher incidence compared to other EGFR-TKIs (Waliany et al., 2021).

Given that osimertinib is the recommended first-line medication for patients with EGFR-mutated NSCLC according to international guidelines (Piper-Vallillo and Sequist, 2019), vigilance against cardiac QT interval prolongation during treatment is crucial, especially for patients with a history of QT prolongation or concurrent use of other QT interval-prolonging medications (Bian et al., 2020; Maimaitituersun et al., 2023). Encouragingly, the predominant cardiac adverse effect of osimertinib has garnered attention from both oncologists and cardiologists. Numerous clinicians have reported cases of QT prolongation induced by EGFR-TKIs treatment, contributing to the evolving understanding of this phenomenon (Oyakawa et al., 2017; Schiefer et al., 2018; Matsuura et al., 2021; Guo et al., 2023).

### 3.2 HF and pericardial effusion

Sarah Waliany et al. selected four EGFR-TKIs in their study, namely gefitinib, erlotinib, afatinib, and osimertinib. Osimertinib exhibited a pronounced impact compared to other EGFR-TKIs, presenting a ROR of 6.75 (99% CI: 5.29–8.62) relative to other NSCLC targeted therapies (ROR = 3.64, 99% CI: 2.94–4.50) and the full database (ROR = 5.37, 99% CI: 4.42–6.54, IC025 = 2.14). Notably, osimertinib has been demonstrated to increase the risk of HF, second only to its risk of QT interval prolongation, with a median onset of 85 days (Waliany et al., 2021).

In 2015, Michael S. Ewer conducted a clinical study on afatinib, indicating no association with HF or decreased LVEF (Ewer et al., 2015). However, in 2019, Giacomo Nuvola et al. reported a case of pericardial effusion following afatinib treatment (Nuvola et al., 2019). Subsequently, in 2020, Saori Ikebe et al. documented a case of concurrent HF and QT prolongation during osimertinib treatment (Ikebe et al., 2020).

In a Phase I study of lapatinib plus trametinib in patients with KRAS-mutant colorectal, non-small cell lung, and pancreatic cancer, a total of 34 patients were enrolled in the study at six different dose levels, including 15 patients with NSCLC. The investigators found that 5 of the 34 patients had decreased LVEF. One patient permanently discontinued study therapy because LVEF dropped from 70% at baseline to 24% after nine cycles and did not improve to more than 50% within 4 weeks of treatment interruption. There was also one patient with a 21% decrease in LVEF who had multiple treatment interruptions (Huijberts et al., 2020).

### 3.3 AF

Wei-Ting Chang et al. conducted a comprehensive analysis of data spanning from 2001 to 2014, focusing on NSCLC patients. Utilizing a Cox proportional risk model, they discerned a significantly elevated risk of ventricular arrhythmia in patients treated with EGFR-TKIs compared to those treated with platinum analogues. Conversely, the risk of AF did not exhibit a significant change relative to patients treated with platinum analogues (Chang et al., 2023).

In a separate investigation, Kartik Anand compared the cardiotoxicity risk of osimertinib with other drugs and various EGFR-TKIs (erlotinib, afatinib, and gefitinib) using the FDA Adverse Event Reporting System (FAERS). The Risk Odds Ratio (ROR) (95% CI) of AF induced by osimertinib was 4.0 (2.8–5.8) compared to all other drugs in FAERS and 2.1 (1.3–3.5) compared to other EGFR-TKIs. These findings suggeste that osimertinib has a higher risk of AF compared to all other drugs in FAERS and other EGFR-TKIs (Anand et al., 2019).

### 3.4 Hypertension

A meticulous systematic review, assessing and contrasting the efficacy and safety of erlotinib combined with bevacizumab *versus* erlotinib monotherapy for advanced NSCLC patients, unveiled noteworthy findings: the amalgamation of erlotinib and bevacizumab showed an increased risk of hypertension (RR = 5.15; 95% CI: 3.59, 7.39; *p* < 0.00001) (Sakharkar and Kurup, 2023). It is well-established that hypertension stands as a prevalent adverse reaction associated with targeted vascular endothelial growth factor receptor inhibitors, comprising both monoclonal antibodies and small molecule kinase inhibitors (Brinda et al., 2016; Yang and Bu, 2016; Chaar et al., 2018; Pantazi and Tselepis, 2022). While the direct causative link between EGFR-TKI drugs in isolation and hypertension remains uncertain, existing evidence suggests the involvement of EGFR/ErbB1 in vascular remodeling (Xiong et al., 2023). Schreier et al. substantiated this by revealing targeted EGFR deficiency in vascular smooth muscle isolated from mouse aortas precipitated spontaneous cell death (Schreier et al., 2011).

### 3.5 MI

In 2005, K. Yamaguchi et al. documented a case of acute MI subsequent to gefitinib treatment, proposing that gefitinib might induce such events by augmenting platelet aggregation (Yamaguchi et al., 2005). In 2015, Tulay Ku et al. reported two instances of acute cardiovascular events associated with erlotinib treatment (Kus et al., 2015). In a retrospective single-center cohort study conducted in 2020 by Kei Kunimasa, the focus was on evaluating osimertinib-related cardiac adverse events within a real-world setting. This study concluded that the incidence of cardiac adverse events in patients undergoing osimertinib treatment was 4.9%, offering a detailed account of a MI case following osimertinib administration (Kunimasa et al., 2020).

### 3.6 Cardiac hypertrophy and cardiomyopathy

In 2016, François Pinquié et al. reported a 71-year-old female patient with metastatic NSCLC who developed dilated cardiomyopathy after 26 months of erlotinib treatment. Despite symptomatic management, the patient’s LVEF dropped to 25% (Pinquié et al., 2016). Concurrently, Hesham M. Korashy employed an *in vitro* rat model to explore the impact of gefitinib on the heart. The study revealed that 21 days of gefitinib treatment (20 and 30 mg/kg) in Wistar albino rats led to an increase in mRNA and protein of B-type natriuretic peptide (BNP) and beta-myosin heavy chain, indicating an increase in the expresssion of hypertrophy genes. Simultaneously, anti-hypertrophic genes were inhibited, accompanied by an increase in the percentage of mast cells (Korashy et al., 2016). In 2020, Abdullah F. AlAsmari corroborated that gefitinib can induce cardiac hypertrophy by using an adult male Wistar rat model. Interestingly, AlAsmari found that the GLP-1 receptor agonist liraglutide demonstrated potential cardioprotective effects against this hypertrophic process (AlAsmari et al., 2020).

In 2022, German E. Ramos et al. reported a clinical case of afatinib inducing Takotsubo cardiomyopathy in an NSCLC patient (Ramos et al., 2022). Subsequently, in 2023, Karishma Patel et al. presented a case of osimertinib-induced biventricular cardiomyopathy, accompanied by cardiac magnetic resonance imaging abnormalities. Following the initiation of goal-directed drug therapy for HF and the retention of osimertinib, the patient’s biventricular function returned to normal. Notably, upon subsequent administration of afatinib, a second-generation EGFR-TKI, there was no recurrence of cardiomyopathy (Patel et al., 2023).

### 3.7 Cardiac valvular lesions

Kei Kunimasa conducted a comprehensive statistical analysis encompassing 123 patients diagnosed with advanced NSCLC and EGFR mutations at the International Cancer Institute, Osaka, spanning the period from 2014 to 2019. Their study showed that 6 patients (4.9%) receiving osimertinib encountered serious cardiac adverse events classified as Common Terminology Criteria for Adverse Events grade 3 or higher. Notably, two of these cases were identified as valvular heart disease (Kunimasa et al., 2020).

## 4 Potential pathways or drugs that protect against the cardiotoxicity of EGFR-TKIs

**FIGURE 3:**
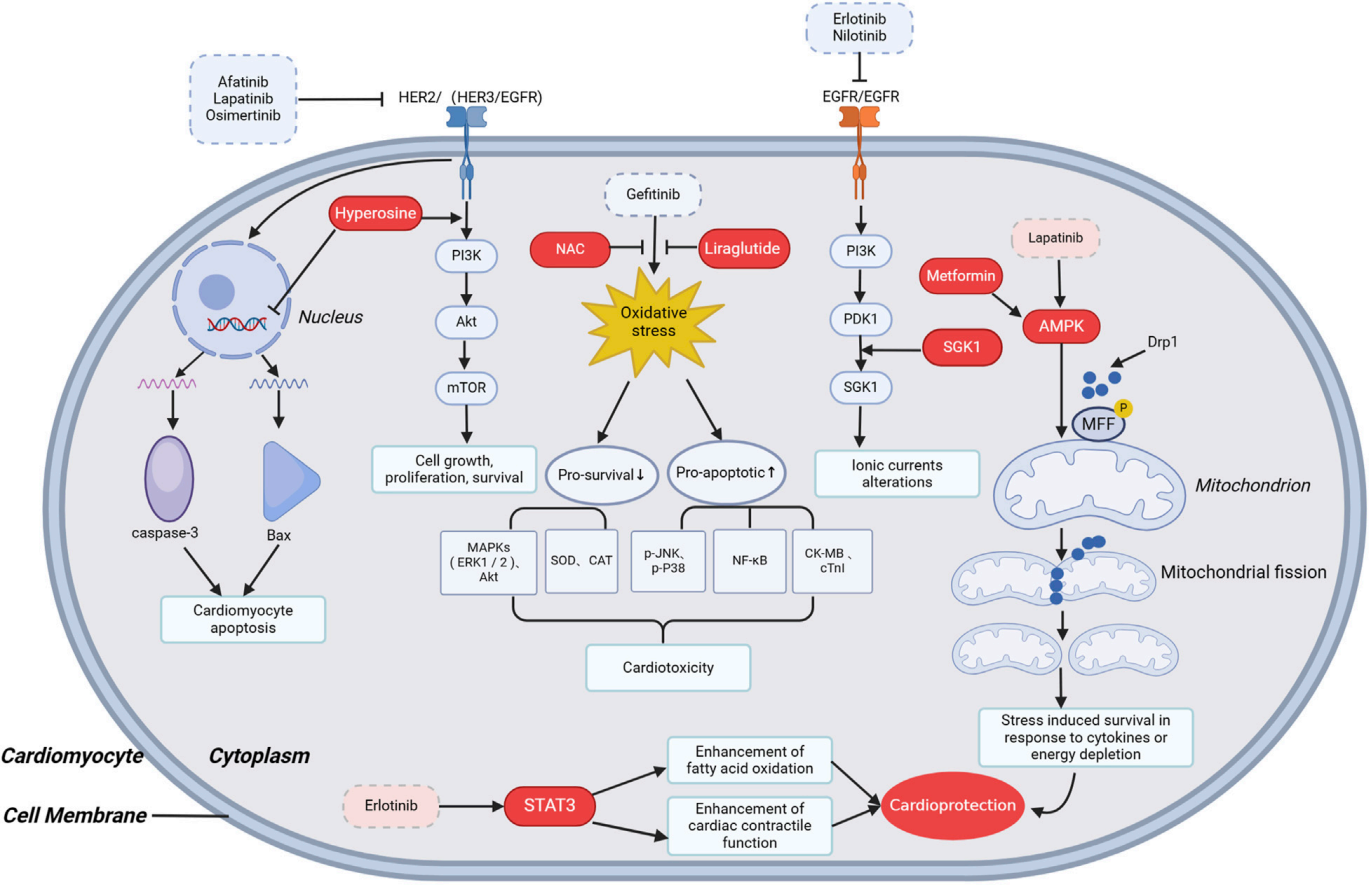
Potential pathways or drugs that protect against the cardiotoxicity of EGFR-TKIs. NAC, N-acetylcysteine; AMPK, Adenosine 5′-monophosphate (AMP)-activated protein kinase; MFF, Mitochondrial fission factor; STAT3, Signal transducer and activator of transcription 3; MAPK, mitogen-activated protein kinase; ERK1/2, extracellular regulated protein kinases1/2; SOD, Superoxide dismutase; CAT, Catalase; p-JNK, p-Jun N-terminal kinase; NF-ĸB, nuclear factor kappa-B; CK-MB, Creatine kinase isoenzymes-MB; cTnI, cardiac troponin I.

According to Xiaoyan Cui et al., the activation of SGK1 demonstrated a counteracting effect on arrhythmias induced by TKIs such as imatinib and erlotinib (Figure 3) (Cui et al., 2022). AMPK (Adenosine 5′-monophosphate (AMP)-activated protein kinase), an AMP-dependent protein kinase, serves as a key regulator of bioenergetic metabolism expressed in various metabolism-associated organs. It can be activated by multiple stimuli, including cellular stress, exercise, and various hormones affecting cellular metabolism (Toyama et al., 2016). The activation of the AMPK pathway by lapatinib may contribute to its comparatively lower cardiotoxicity compared to other TKIs (Shell et al., 2008). Other AMPK activators, such as metformin, have been demonstrated to have protective effects against several cardiac conditions (Figure 3) (Bromage and Yellon, 2015).

Erlotinib, a highly selective EGFR inhibitor with low cardiotoxicity risk, is associated with upregulation of the JAK/STAT pathway in mouse models, potentially providing cardioprotection (Figure 3) (Stuhlmiller et al., 2017). *In vivo* and cellular studies indicated that erlotinib treatment led to STAT3 activation, a phenomenon not observed with sorafenib and sunitinib treatment. Co-administration of erlotinib with STAT3 inhibitors resulted in a decrease in systolic function (Stuhlmiller et al., 2017).

In an experimental rat model, both metoprolol and diltiazem significantly prevented the QT-prolonging effect of pazopanib (Akman et al., 2014). Similarly, Chia-Hao Chang conducted a retrospective cohort study and found that prior β-blocker use was associated with a better prognosis in treatment-naive advanced lung adenocarcinoma patients treated with first-line EGFR-TKIs (Chang et al., 2020).

Based on a self-developed and validated computational model of cardiomyocyte apoptosis, Grabowska’s team performed network simulations of the combination treatment of sorafenib and the antioxidant N-acetylcysteine (NAC), showing that NAC protected cardiomyocytes from sorafenib-induced apoptosis. According to this, they hypothesized that ROS inhibition by NAC or other antioxidants would reduce cell apoptosis induced by TKIs such as sunitinib, ponatinib, trastuzumab, gefitinib, nilotinib, and erlotinib (Figure 3) (Grabowska et al., 2021).

According to the research conducted by Abdullah F. AlAsmari et al., gefitinib treatment resulted in an increase in oxidative stress genes, a decrease in antioxidant proteins, and a decrease in pro-survival kinases, whereas pretreatment with the GLP-receptor agonist liraglutide attenuated these effects and was cardioprotective through the activation of pro-survival kinases (Figure 3) (AlAsmari et al., 2020).

As we know, trastuzumab is a representative drug targeting HER2. Shanshan Wei et al. recently demonstrated that trastuzumab downregulated the PI3K/Akt signaling pathway in mice and H3c9 cells. Combination therapy with hypericin can effectively increase the p-Akt/Akt ratio, thereby inhibiting trastuzumab-induced cardiotoxicity (Figure 3) (Wei et al., 2023). These findings encouraged further prospective clinical studies to validate the potential of the above pathways or drugs as adjuvant anticancer therapy to attenuate cardiotoxicity.

## 5 Risk assessment

Cancer and cardiovascular disease share many common risk factors, with cancer itself increasing the risk for cardiovascular events (Anderson and Sawyer, 2008; Lyon et al., 2020). A comprehensive cardiovascular assessment is therefore crucial before initiating cardiotoxic EGFR-TKIs, ideally at cancer diagnosis and before starting treatment. This allows oncology teams to integrate cardiovascular risk into treatment decisions, undertake personalized monitoring and follow-up, establish appropriate treatment protocols, and refer high-risk patients to cardiac oncology services. It is crucial to emphasize that continuous cardiovascular monitoring during treatment is also vital to prevent serious adverse events (Lyon et al., 2022).

Generally, the baseline cardiovascular risk assessment for tumor patients may include the following components: clinical history (including past medical history), physical examination, serum cardiac markers, electrocardiogram (ECG), cardiac imaging, cardiovascular risk factor screening, and cardiopulmonary fitness assessment (Curigliano et al., 2016; Lyon et al., 2022). The medical history should be thorough, as previous cardiovascular conditions, chronic kidney disease, and genetic factors can elevate the risk of cardiovascular disease during treatment (Lyon et al., 2020). Physical examinations should record vital signs and identify potential signs of undiagnosed cardiovascular disease such as HF, pericardial disease, and arrhythmias. Common cardiac markers include troponin, high-sensitive cardiac troponin, BNP, and NT-proBNP. Research indicates that in patients treated with antitumor drugs, troponin can detect cardiotoxicity at a preclinical stage, well before any decrease in LVEF occurs (Cardinale et al., 2010). A baseline 12-lead ECG, an accessible and routine test, provides vital insights into potential cardiovascular diseases. The Fridericia correction (QTcF) is recommended to measure the QT interval (Porta-Sánchez et al., 2017). In the general population, the upper normal limit for QTc values is 450 ms for men and 460 ms for women, with QTc≥500 ms associated with a two-to three-fold increased risk of TdP (115). Although the incidence of QTc≥500 ms and TdP in cancer treatment is low, extending the QTc to ≥480 ms necessitates closer clinical monitoring (Zamorano et al., 2016; Herrmann, 2020; Salem et al., 2021). Echocardiography, particularly three-dimensional (3D), is the preferred method for assessing LVEF and cardiac function. LVEF below 50% is considered a risk factor for future cardiovascular diseases in most cardiotoxic cancer therapies and should warrant physician attention. When 3D ultrasound is unavailable, two-dimensional ultrasound may be considered (Santoro et al., 2017; Zhang et al., 2018; Čelutkienė et al., 2020). If ultrasound image quality is inadequate, cardiac MRI is recommended if available (Wu et al., 2009; Plana et al., 2018; Dobbin et al., 2020).

Key cardiovascular risk factors include hypertension (blood pressure >140/90 mmHg), diabetes (fasting blood glucose >126 mg/dL or HbA1c >6.5%), dyslipidemia (total cholesterol >240 mg/dL, LDL cholesterol >160 mg/dL, HDL cholesterol <40 mg/dL), obesity, smoking, sedentary lifestyle, advanced age, and others (Sharifi-Rad et al., 2020; Cheng et al., 2021). The 2022 ESC Guidelines recommend a three-monthly echocardiograph for patients taking osimertinib and advise lipid monitoring at least twice a year (Lyon et al., 2022). Additionally, factors such as timing and method of administration, potential drug interactions, individual susceptibility (including genetic abnormalities), female gender, bradycardia, electrolyte imbalances (hyper/hypokalemia, hypomagnesemia, hypocalcemia), hypothermia, hypothyroidism, and pre-existing medical conditions all contribute to the prolonged QT interval and TdP risk of drug response (Roden, 2016; Zamorano et al., 2016; Curigliano et al., 2020; Kaira et al., 2020). These factors should be thoroughly considered in the cardiac risk assessment before and during treatment with EGFR-TKIs.

## 6 Management and countermeasures of cardiotoxicity induced by EGFR-TKIs

Currently, there is no global consensus on the specific issue of management and countermeasures of cardiotoxicity caused by EGFR-TKIs. However, existing research and clinical experience suggest that comprehensive preventive measures, monitoring, treatment adjustments, and multidisciplinary collaboration are crucial in managing cardiotoxicity induced by EGFR-TKIs.

Educating patients to recognize and report cardiac symptoms like palpitations, chest pain, and dyspnea is crucial (Lyon et al., 2022). Medical professionals must act swiftly and appropriately when monitoring indicators show abnormalities during treatment. While cardiotoxic effects induced by EGFR-TKIs are often reversible and non-fatal after discontinuing or adjusting the medication, immediate attention is needed for life-threatening complications such as prolonged QT interval and ventricular arrhythmia. Treatment should be paused for patients with a QTc interval over 500 milliseconds on consecutive ECGs until the QTc interval falls below 481 milliseconds or returns to baseline (if baseline is ≥ 481 milliseconds), after which medication can be cautiously resumed at a reduced dose. Permanent discontinuation is necessary for patients with prolonged QTc who exhibit symptoms or signs of torsades de pointes, polymorphic ventricular tachycardia, or severe arrhythmias (Virani et al., 2016; Coppola et al., 2018; Guo et al., 2023). Despite a lack of formal consensus, clinical experience supports the efficacy of interventions like ventricular pacing and supplementation with potassium and magnesium in shortening the QT interval and terminating TdP (Fradley et al., 2021; Zeppenfeld et al., 2022; Zhang et al., 2022).

Regular echocardiography monitoring is essential, with LVEF being a crucial indicator that should ideally be above 55% for healthy individuals. Should LVEF decrease by more than 10% from baseline or fall below 50%, it is recommended to reduce, switch, or temporarily halt EGFR-TKI treatment until cardiac indicators improve (Virani et al., 2016; Lyon et al., 2020; Lyon et al., 2022). Hypertension, a prevalent cardiovascular risk factor and a possible side effect of EGFR-TKIs, demands serious attention from clinicians. For patients with cardiovascular risk factors, antihypertensive treatment should commence if blood pressure (BP) > 130/80 mmHg; for others, intervention should start at BP > 140/90 mmHg, with ACE inhibitors or ARBs as the first-line treatment. When BP > 180/110 mmHg, consideration should be given to stopping or changing cancer-targeted therapy until BP is controlled<140/90 mmHg (Lyon et al., 2022). Cancer treatment can resume, potentially at a reduced dosage, once BP stabilizes. Additionally, factors like stress, pain, excessive alcohol intake, renal impairment, untreated sleep apnea, obesity, and reduced physical activity, which can influence blood pressure, should be addressed (Jaspan et al., 2024).

Beyond these strategies, cardiac rehabilitation, which includes aerobic exercise, strength training, and educational activities, is vital for enhancing cardiac health and overall patient wellness (Viamonte et al., 2024). The emotional and psychological support provided by mental health professionals is also crucial. Finally, the effective management and prevention of cardiotoxicity associated with EGFR-TKIs rely on the collaboration among cardiologists, oncologists, pharmacists, and rehabilitation specialists. This multidisciplinary approach ensures the safety and effectiveness of the treatment and maximizes the quality of life and treatment outcomes for patients.

## 7 Future prospects

Future perspectives on EGFR-TKI-induced cardiotoxicity encompass more in-depth investigations and refined monitoring methods to comprehensively understand and manage this potential risk. As medical science advances, we anticipate more precise molecular biology and genetics studies that hold the promise of identifying individual characteristics predisposing patients to cardiotoxicity from EGFR-TKIs. Adopting an individualized medicine approach could empower physicians to better foresee a patient’s cardiac response and implement suitable measures, such as adjusting drug dosages or selecting alternative treatment options.

Furthermore, upcoming studies may delve into novel drug combinations or interventions aimed at mitigating the risk of cardiotoxicity associated with EGFR-TKIs, thus enhancing patient outcomes and overall survival. However, the realization of these advancements will need continuous clinical trials and rigorous monitoring to ascertain the safety and efficacy of these innovative strategies. In conclusion, the future outlook for EGFR-TKI-induced cardiotoxicity involves personalized treatment approaches, improved risk prediction, and more effective therapeutic strategies, ultimately enhancing the quality of life and survival prospects for individuals with EGFR-mutated cancers.

## 8 Conclusions

EGFR-TKIs currently stand out as the most effective and comparatively well-tolerated drugs for clinically treating patients with EGFR mutations. However, their use is accompanied by a significantly elevated risk of cardiotoxicity compared to traditional chemotherapeutic agents like platinum. This heightened risk often necessitates the reduction or discontinuation of EGFR-TKI treatment during targeted therapy, ultimately leading to tumor progression. Presently, the mechanisms underlying cardiotoxicity induced by EGFR-TKIs remain incompletely understood, and the distinctions in cardiotoxicity mechanisms among different EGFR-TKIs remain unclear.

Consequently, extensive research is imperative to unravel these mechanisms in the future. This endeavor is crucial not only for enhancing our understanding of the intricacies of EGFR-TKI-induced cardiotoxicity but also for providing valuable insights into the development of new drugs. Moreover, such insights can contribute to the formulation of preventive or therapeutic strategies for managing cardiac damage in patients undergoing clinical treatment with EGFR-TKIs. The pursuit of these research avenues holds the potential to advance both the safety profile of existing therapies and the development of novel interventions in the clinical landscape.
